# Effects of *Saccharomyces boulardii* cell wall polysaccharide supplementation on growth performance, serum immunity, and fecal microorganisms in newborn calves

**DOI:** 10.3389/fvets.2025.1543859

**Published:** 2025-05-12

**Authors:** Haina Yin, Xucheng Mo, Wenshuai Zeng, Wenshu Zhu, Mengjian Liu

**Affiliations:** ^1^College of Animal Science, Xinjiang Agricultural University, Urumqi, China; ^2^Animal Health Supervision Station of Changji National Agricultural Science and Technology Park, Changji, China

**Keywords:** *Saccharomyces boulardii* wall polysaccharide, newborn calves, growth performance, serum immunity, intestinal microbiota

## Abstract

**Background:**

Xinjiang is characterized by extremely cold weather and significant seasonal temperature variations, and these harsh climatic conditions have led to a high incidence of diarrhea and increased mortality rates among newborn calves, resulting in substantial economic losses for the local cattle industry. *Saccharomyces boulardii* cell wall polysaccharide (SBWP) is a natural prebiotic that has emerged as a promising alternative to conventional antibiotics for the mitigation of systemic inflammation, diarrhea, and mortality in livestock production. Therefore, this study aimed to investigate the effects of SBWP supplementation on growth performance, diarrhea frequency, serum immunity, and intestinal microbiota in newborn calves.

**Methods:**

In this study, a one-way experimental design was employed. A total of 45 newborn calves (Simmental♂ × Yili brown cattle♀, male, average body weight (BW) 35.58 ± 5.79 kg) were randomly allocated into five experimental groups. Each group consisted of three pens, with three calves per pen, and this allocation was carried out based on the percentage of SBWP used as a feed supplement. The diet for the five groups were as follows: group I received milk + basal diet without additives, group II received milk + basal diet + 0.005% gentamicin, group III received milk + basal diet + 250 mg/day/calf SBWP, group IV received milk + basal diet + 500 mg/day/calf SBWP, and group V received milk + basal diet + 1,000 mg/day/calf SBWP. Daily feed consumption was recorded, and BW was measured on days 1, 14, and 28 to calculate average daily gain (ADG), average daily feed intake, and feed-to-gain (F/G) ratio. Fecal samples were collected on days 1, 7, 14, 21, and 28 for microbiological analysis, and fecal scores were subjectively monitored and recorded daily by the same individual. In addition, blood samples were collected from each calf at the end of the trial for immune analysis.

**Results:**

In comparison to group I, group IV showed a significant increase in both BW and ADG. Specifically, on the 14th and 28th trial days, BW of group IV showed a significant increase of 3.95 and 4.90%, respectively (*p* < 0.05). Similarly, during the 1–28 trial day period, ADG of group IV showed a significant increase of 28.49% (*p* < 0.05), whereas their F/G ratio decreased significantly by 22.89% (*p* < 0.05). No statistically significant difference was observed in BW, ADG, dry matter intake, and F/G ratio (*p* > 0.05) between groups IV and II. In addition, the fecal score and the diarrhea rate in group IV were significantly reduced by 31.62 and 18.54%, respectively (*p* < 0.05). No statistically significant difference was observed between groups IV and II (*p* > 0.05). Moreover, in group IV, IgG and IL-10 levels were significantly increased by 51.97 and 45.45%, respectively (*p* < 0.05), while IL-1, IL-6, and TNF-*α* levels were significantly decreased by 30.47, 28.17, and 25.49%, respectively (*p* < 0.05). Furthermore, a decreasing trend in the number of *Escherichia coli* and *Clostridium perfringens* was observed in the fecal microbiota samples obtained from group IV, whereas an increasing trend was observed in the growth of *Lactobacillus* and *Bifidobacterium*. Supplementing newborn calves with 500 mg of SBWP per day significantly enhanced the *β*-diversity indices and demonstrated a trend toward increasing *α*-diversity in their fecal microbiota, in contrast to the detrimental effects caused by 0.005% gentamicin. Furthermore, 500 mg/day/calf SBWP significantly increased the relative abundance of *Lactobacillus* and decreased the relative abundance of *Escherichia-Shigella*. However, no significant difference was observed in the relative abundance of *Escherichia-Shigella* between the groups receiving 500 mg/day/calf SBWP and 0.005% gentamicin.

**Conclusion:**

The findings of this study show that the supplementation of 500 mg/day/calf SBWP to newborn calves significantly improves their growth performance, serum immunity, and intestinal microbiota structure while significantly reducing diarrhea frequency and inflammation. These findings indicate that the supplementation of 500 mg/day/calf SBWP can most effectively enhance the growth performance and reduce the diarrhea frequency in newborn calves.

## 1 Introduction

Newborn calves face critical health challenges due to their immature immune system and gut barrier, rendering them highly susceptible to intestinal pathogens and environmental stressors (e.g., temperature fluctuations and dietary transitions) (1). In Northern Xinjiang’s mountain valley pastoral systems (800–1,200 m altitude, temperate continental climate of 6.5–8.5°C annual temperature, 400–600 mm precipitation), these risks are exacerbated due to seasonal management practices (summer grazing + winter stall-feeding) involving extreme diurnal temperature variation (10–15°C) and abrupt forage shifts (2). These challenging conditions can trigger gastrointestinal dysfunction and diarrhea in newborn calves, with mortality rates as high as 15% (3), thus posing a major obstacle to sustainable beef production in the region.

Existing strategies depend heavily on antibiotic prophylaxis, but this practice disrupts gut microbiota maturation, impairs mucosal immunity, and induces antimicrobial resistance. There is an urgent demand for antibiotic alternatives that enhance intestinal health *in situ*. *Saccharomyces boulardii* cell wall polysaccharide (SBWP), a prebiotic macromolecule composed of mannose and *β*-glucan, offers a promising solution. Studies have shown that SBWP enhances immunity by directly interacting with intestinal mucosal epithelial cells via pathogen-associated molecular patterns (PAMPs) (3). Mannose and *β*-glucan mediate immune cell signaling primarily through phagocytic pattern recognition receptors: mannose receptor (MR) of the C-type lectin receptor (CLR) family for mannose and DC-associated C-type lectin-1 (dectin-1 receptor) for β-glucan. Both compounds also use membrane-bound Toll-like receptors for cellular signal transduction (4, 5). In addition, SBWP modulates intestinal microbiota and metabolites by acting as a probiotic and adsorbing pathogenic bacteria and toxins (6). These characteristics make SBWP a widely used and extensively studied additive. Although SBWP improves growth and immunity in weaned calves (7), its effects in *newborn* calves (0–28 days) remain underexplored. This critical neonatal window is characterized by (1) rapid gut microbiota colonization (8), (2) mucosal immune ontogeny (9), and (3) high sensitivity to environmental stressors. The mannose-rich structure of SBWP may specifically interact with neonatal gut lectin receptors and promote mucosal barrier maturation (10). Furthermore, its *β*-glucan moiety activates neonatal dendritic cell dectin-1, which primes Th1/Th17 responses that are crucial for clearing pathogen (11).

In this study, newborn Simmental×Yili Brown crossbred calves from the northern pastoral region of Xinjiang were used. The effects of SBWP supplementation on growth performance, average fecal score, and diarrhea frequency of the newborn calves were evaluated. In addition, the impact of SBWP on serum immunity and fecal microbiota composition in newborn calves was investigated. Based on the results, the optimal dosage of SBWP was determined, providing theoretical support for its application in calf production.

## 2 Materials and methods

All experimental protocols were conducted in compliance with the ethical guidelines approved by the Institutional Animal Care and Use Committee (IACUC) of Xinjiang Agricultural University, College of Veterinary Medicine. The study adhered to the Animal Research: Reporting of *In Vivo* Experiments (ARRIVE) guidelines to ensure the reproducibility and ethical integrity of the research.

The experimental trials were carried out at a livestock cooperative located in Yili, China (geographical coordinates: 81°34′–83°35′E, 42°54′–43°38′N) from 6 November to 4 December 2024. At the trial commencement, daytime temperatures ranged from approximately 5 to 10°C and nighttime temperatures from −5 to 0°C. By the trial conclusion, daytime temperatures decreased to 0–5°C and nighttime temperatures to −10−−5°C. The experimental site is situated at an average altitude of 3,500 m above sea level, with a mean annual temperature of 2.9°C.

### 2.1 Preparation of SBWP

SBWP was obtained through a series of biotechnological processes, including microbial fermentation, solvent extraction, and chromatographic purification, which were conducted following the conditions mentioned in a previous study (8). The optimal parameters for ultrasound-assisted extraction were as follows: NaOH addition 52.63%, ultrasonic power 143.15 W, and ultrasonic time 86.20 min, which resulted in an optimized extraction yield of 37.54%. The molecular weight of the primary components BLC-1 and BLC-2 in SBWP was 164.68 KDa and 13.21 KDa, whose proportion in SBWP was 24.57 and 66.08%, respectively. The proportion of glucose and mannose was 47.68 and 39.18% in BLC-1 and 76.59 and 6.86% in BLC-2, respectively (8). The nutritional profile of SBWP was quantitatively analyzed using standardized methods, which resulted in the following findings: polysaccharides (84%), crude protein (4%), crude fat (3%), crude ash (2%), and moisture content (7%), following Chinese National Standards GB/T 6434–94, GB/T 6432–94, and GB/T 6435–86. Gentamicin sulfate, which was used as a comparative antibiotic, was obtained from Hebei Jiupeng Pharmaceutical Co., Ltd., in a soluble powder form with a potency of 8 million IU/g.

### 2.2 Animal, diets, and experiment design

A total of forty-five healthy newborn male crossbred calves (Simmental male×Yili brown cattle female) with an average body weight (BW) of 35.58 ± 5.79 kg were randomly allocated to five experimental groups using a completely randomized design, with three replicates per group (n = 3 calves/replicate). The dietary treatments were as follows: group I (control group), milk + basic diet without supplementation; group II, milk + basic diet with 0.005% gentamicin sulfate; group III, milk + basic diet with 250 mg/day/calf weight of SBWP; group IV, milk + basic diet with 500 mg/day/BW of SBWP; and group V, milk + basic diet with 1,000 mg/day/BW of SBWP. The milk replacer was prepared according to the nutritional specifications outlined in the China Beef Cattle Breeding Standard (Table 1).

**Table 1:** Composition and nutrient levels of basal diet (air-drying basis).

Ingredients	Content (%)	Nutrient levels	Content (%)
Basal diet		Total energy (MJ/kg)	16.84
Soybean meal	25	Digestible energy (DE) (MJ/kg)	12.51
Extruded full-fat soybean	13	Crude protein (CP)	19.95
Whey powder	5	Ether extract (EE)	4.66
Corn	25	Crude fiber (CF)	5.5
Expanded corn	17.9	Acid detergent fiber (ADF)	6.03
Wheat bran	10	Neutral detergent fiber (NDF)	16.54
Calcium hydrogen phosphate	0.8	Ash	5.4
Sodium chloride	0.5	Calcium	1.16
Limestone	1.8	Phosphorus	0.59
Premix	1	Lysine	1.52
Total	100	Methionine + cysteine	0.96

^1Without adding any antibiotic to the feed ingredients.^

^2^The premix provided the following milk replacer (per kilogram): VA 10000 IU, VB_1_ 3 mg, VB_2_ 2.5 mg, VB_5_ 4 mg, VB_6_ 2.3 mg, VB_12_ 0.01 mg, VD_3_ 1,000 IU, VE 20 IU, Fe 40 mg, Mn 20 mg, Cu 6 mg, Zn 30 mg, I 0.2 mg, Se 0.3 mg.

^3The nutrient levels were calculated values.^

### 2.3 Animal husbandry and management

Each group consisted of three pens with three calves per pen, which were bedded with 2–3 cm of straw. During the experimental period, due to low temperatures, especially nighttime temperatures dropping below 0°C, thermal insulation measures were implemented for both milk and drinking water in all pens to prevent the freezing of water and large-scale calf diarrhea. Standardized feeding protocols and immunization schedules were strictly followed in accordance with the farm’s operational procedures.

Newborn calves were provided 4 L of bovine colostrum within 2 h post-parturition. From days 0 to 8, calves were fed 4–8 L of warm milk (36–38°C) eight times daily, with a daily increment of 0.5 L. During days 8–28, the feeding regimen was adjusted to 8–12 L of milk at the same temperature, administered four times daily. Milk was dispensed in 1-L sterile-sealed bottles premixed with the respective SBWP concentrations. A basal diet was introduced at 7 days postpartum, starting at 20 g/day and increasing by 10 g/day thereafter. Alfalfa hay and temperature-controlled water were provided *ad libitum* throughout the study.

Newborn calves were subjected to colostrum management (≥4 L of maternal colostrum within 6 h of birth, containing ≥50 g/L IgG) to establish passive immunity. Active immunization included subcutaneous administration of multivalent vaccines (rotavirus, coronavirus, *Clostridium perfringens* types C/D) 24–48 h after birth, followed by booster doses at 2 and 4 weeks of age.

### 2.4 Sample collection

Fecal samples (20 g) were aseptically collected from each calf on experimental days 1, 7, 14, 21, and 28. Samples from each replicate were thoroughly mixed and immediately preserved in sterile collection bags containing ATCC-recommended cryoprotectant solution (10% glycerol + 5% dimethyl sulfoxide + culture medium). Then, they were flash-frozen in liquid nitrogen and stored at −196°C for subsequent microbiological analysis within 48 h.

All blood samples were randomly collected in 10-ml tubes at the end of the study from each newborn calf via cervical venipuncture. Each tube containing the blood sample was centrifuged at 5000 rpm for 15 min at 4°C, and serum was stored at −80°C for immunoassay measurements (9).

### 2.5 Analytical methods

#### 2.5.1 Growth performance parameters

Milk consumption, basal diet intake, and alfalfa hay consumption were measured daily to calculate dry matter intake (DMI) for the three experimental periods: days 1–14, 15–28, and the entire 28-day trial. The BW of newborn calves was recorded on the morning of trial days 1, 14, and 28, and the average daily gain (ADG) and feed gain (F/G) ratio were determined as follows:


$$\begin{array}{l} \mathrm{Average daily feed intake}\;\left(\mathrm{ADFI}\right)=\\ \mathrm{total feed intake}/\mathrm{experimental days}\text{.} \end{array}$$

ADG=total weight gain/experimental days.

$$\mathrm{Feed conversion ratio}\;\left(FCR\right)=ADG/\mathrm{ADFI}\text{.}$$

#### 2.5.2 Determination of fecal score and diarrhea frequency

A single trained observer evaluated fecal characteristics daily using a standardized scoring system. As shown in Table 2, a hierarchical system of average fecal score was established, which was used to indicate the extent of the severity and the presence of diarrhea. When the fecal score was higher than 3, it was counted as one diarrhea day (10). When the fecal score of a calf was higher than 3 and sustained for two consecutive days, it was recorded as one diarrhea calf. At the end of the trial, the average fecal score and average diarrhea frequency were calculated as follows:


$$\begin{array}{l} \mathrm{Average fecal score}\;\left(\%\right)=\\ \left[\Sigma \mathrm{fecal scores}/\mathrm{the total number of calves}\right]\times 100\%\text{.} \end{array}$$

$$\begin{array}{l} \mathrm{Diarrhea frequency}\;\left(\%\right)=[\mathrm{number of diarrhea calves}\times \mathrm{diarrhea days})\\ /(\mathrm{the total number of calves}\times \mathrm{experimental days}]\times 100\%\text{.} \end{array}$$

**Table 2:** A hierarchical system of average fecal score.

Score	Trait
1	Hard, firm feces with a definite shape and dark brown color, without a fetid odor.
2	Slightly soft feces with a definite shape and dark brown color, without a fetid odor.
3	Partially formed feces with a loose shape and dark yellow color, with a light fetid odor.
4	Semiliquid feces with a coagulum and faint yellow, white color, with a distinct fetid odor.
5	Watery, mucous-like feces with blood or mucus and faint yellow, white, and grey color, with a pungent fetid odor.

#### 2.5.3 Determination of serum immune factors

The serum samples were defrosted at room temperature (25°C). Serum concentrations of IgG, IL-1, IL-6, IL-10, TNF-*α*, and IFN-*γ* were measured using assay kits in accordance with the standard procedures (Nanjing Jian Cheng Bioengineering Institute, Nanjing, China) (11).

#### 2.5.4 Determination of four species of fecal bacteria

The fecal samples were defrosted on a sterile table at room temperature (25°C), and 5 g of the samples was accurately weighed, mixed with 45 mL 0.9% sodium chloride solution in a sterile beaker, and stirred for 5 min. *Lactobacillus*, *Bifidobacterium*, *Escherichia coli*, and *C. perfringens* were detected using the plate counting method, and their dilution factors reached 10^−6^, 10^−5^, 10^−5^, and 10^−3^, respectively. The total number of colonies was expressed in CFU/mL. The culture mediums and conditions are shown in Table 3.

**Table 3:** Culture mediums and conditions of different bacteria.

Bacterial species	Medium	Condition
*Lactobacillus*	MRS medium	Anaerobic culture, 42°C, pH = 6.3, 48 h
*Bifidobacterium*	BBL medium	Anaerobic culture, 37°C, pH = 7.0, 48 h
*Escherichia coli*	Eosin methylene blue medium	Aerobic culture, 37°C, pH = 7.2, 48 h
*Clostridium perfringens*	Modified SPS medium	Anaerobic culture, 37°C, natural pH, 48 h

### 2.6 Analysis of fecal microbiota diversity and structure

Six 28-day fecal samples each from groups I, II, and IV were randomly selected and thawed at ambient temperature (25°C). Genomic DNA was extracted from 1.0 g of fecal samples using the QIAamp DNA Stool Mini Kit (Qiagen, Hilden, Germany). Integrity and purity of the extracted DNA were verified, and the qualified DNA samples were used as templates for polymerase chain reaction (PCR) amplification of V3–V4 hypervariable regions of the bacterial 16S ribosomal RNA (rRNA) gene. PCR amplification was carried out using barcoded primers 341F (5’-CCTAYGGGRBGCA SCAG-3′) and 806R (5’-GGACTACNNGGGTATCTAAT-3′). The resulting PCR amplicons were purified and subsequently used to construct sequencing libraries. These libraries were then subjected to paired-end sequencing on the Illumina MiSeq platform (Illumina Inc., San Diego, CA, USA) following the standard protocols provided by Novogene Bioinformatics Technology Co., Ltd. (Beijing, China). Raw sequence data were subjected to quality control processing using the UCHIME algorithm to generate high-quality clean reads. These filtered reads were then clustered into operational taxonomic units (OTUs) using Uparse software (version 7.0, https://drive5.com/uparse/), with a 97% sequence similarity threshold. Representative sequences from each OTU were selected for taxonomic classification using the Mothur algorithm against the SILVA rRNA database. Microbial *α*-diversity indices, including OTU richness, Shannon diversity index, Simpson diversity index, and Chao1 estimator, were calculated using QIIME 2 software. Furthermore, *β*-diversity analysis was carried out based on unweighted UniFrac distances, and the results were visualized using principal coordinate analysis (PCoA).

### 2.7 Statistical analysis

Primary data were recorded and processed using Microsoft Excel (version 2022, Microsoft Corp., USA). Statistical analyses were carried out using SPSS Statistics (version 18.0, IBM Corp., USA). Data were expressed as mean ± standard deviation (SD). One-way ANOVA followed by Duncan’s multiple range test was used to determine significant differences between treatment groups at *p* < 0.05.

## 3 Results

### 3.1 Effect of SBWP on growth performance of newborn calves

As shown in Table 4, no significant differences (*p* > 0.05) were observed in the initial BW of newborn calves across the experimental groups, which confirms the appropriateness of the experimental design. Compared with group I (control), group IV showed a significant increase in BW by 3.95% (*p* < 0.05) and 4.90% (*p* < 0.05) on trial days 14 and 28, respectively. Significant differences in ADG were observed between the groups, with group IV showing a 28.49% improvement (*p* < 0.05) over group I during the 1–28 trial day period. Similarly, the F/G ratio was significantly reduced by 22.89% (*p* < 0.05) in group IV compared with group I during the same period. In addition, BW and ADG of group IV showed no significant difference from those of group I (*p* > 0.05). Notably, no significant differences (p > 0.05) were observed in ADFI between the groups. These findings indicate that supplementation with 500 mg/d/Calf SBWP significantly enhances ADG and reduces F/G ratio without significantly altering ADFI in newborn calves.

**Table 4:** Effects of SBWP on growth performance of newborn calves.

Items	Time	Group I	Group II	Group III	Group IV	Group V
BW (Kg)	1 d	35.82 ± 1.12	35.22 ± 3.02	35.72 ± 1.96	35.48 ± 1.30	35.62 ± 1.19
	14 d	38.69 ± 1.16^a^	40.16 ± 0.3^b^	39.32 ± 1.40^ab^	40.22 ± 1.25^b^	40.07 ± 1.79^b^
	28 d	44.70 ± 1.34^a^	46.94 ± 2.66^b^	46.11 ± 1.59^ab^	46.89 ± 1.22^b^	46.63 ± 1.79^b^
ADG (g/d)	1–14 d	204.84 ± 32.14	352.38 ± 196.43	257.14 ± 53.57	338.89 ± 18.29	317.46 ± 55
	15–28 d	429.36 ± 32.86	484.92 ± 168.57	485.34 ± 25.00	476.19 ± 46.42	469.05 ± 64.26
	1–28 d	317.14 ± 19.29^a^	418.57 ± 39.29^b^	371.07 ± 26.43^b^	407.50 ± 26.43^b^	393.21 ± 41.07^b^
ADFI (g/d)	1–14 d	772.48 ± 38.07	746.20 ± 30.78	750.62 ± 10.77	744.99 ± 33.14	743.39 ± 51.56
	15–28 d	1021.81 ± 79.02	1009.46 ± 90.66	1014.19 ± 75.05	1006.5 ± 87.75	1013.38 ± 27.31
	1–28 d	897.14 ± 23.52	881.18 ± 14.68	877.43 ± 23.53	875.74 ± 15.39	891.13 ± 47.76
F/G	1–14 d	3.79 ± 0.24	2.11 ± 0.11	2.93 ± 0.14	2.20 ± 0.18	2.33 ± 0.21
	15–28 d	2.38 ± 0.31	2.09 ± 0.18	2.09 ± 0.15	2.12 ± 0.13	2.16 ± 0.09
	1–28 d	2.84 ± 0.33^b^	2.10 ± 0.14^a^	2.34 ± 0.21^a^	2.19 ± 0.09^a^	2.26 ± 0.14^a^

BW, body weight; ADG, average daily gain; ADFI, average daily feed intake; F/G, feed-to-gain ratio.

Values with no superscript letter or the same superscript letter indicate no significant difference (*P* > 0.05), whereas those with different superscript letters indicate a significant difference (*P* < 0.05). The same below.

### 3.2 Effect of SBWP on diarrhea incidence in newborn calves

As shown in Table 5, group II (gentamicin-supplemented group) showed the lowest fecal score of 1.70, which was significantly lower than that of group I (*p* < 0.05). Fecal scores of groups IV and V were 1.73 and 1.76, respectively, representing significant reductions of 31.62 and 31.04% (*p* < 0.05) compared with group I. However, the difference in fecal scores between groups IV and II was not significant (*p* > 0.05). Similarly, group II showed the lowest diarrhea incidence rate (12.16%), which was significantly lower than that of group I (*p* < 0.05). Diarrhea rate of groups IV and V was 14.02 and 14.55%, respectively, representing reductions of 18.54 and 15.46% compared with group I. No significant difference in fecal scores (*p* > 0.05) was observed between groups IV and II. These results show that 0.005% gentamicin supplementation is the most effective in preventing diarrhea. However, supplementation with 500 mg/d and 1,000 mg/d SBWP also effectively mitigated diarrhea, with no significant difference (*p* > 0.05) compared with gentamicin supplementation.

**Table 5:** Effect of SBWP on diarrhea in newborn calves.

Items	Group I	Group II	Group III	Group IV	Group V
Diarrhea score	2.53 ± 0.18^c^	1.70 ± 0.13^a^	2.24 ± 0.22^b^	1.73 ± 0.10^a^	1.76 ± 0.06^a^
Diarrhea rate (%)	17.21 ± 3.17^b^	12.16 ± 1.54^a^	17.38 ± 3.59^ab^	14.02 ± 1.86^a^	14.55 ± 1.33^a^

### 3.3 Effect of SBWP on serum immune factors in newborn calves

As shown in Table 6, serum IgG level in group IV was significantly higher (17.79 mg/g) than in groups I and II (*p* < 0.05). Compared with group I, IgG level in groups IV and V increased by 51.97 and 48.61% (*p* < 0.05), respectively. Group II showed the lowest IL-1 level (2.45 mg/g), which was significantly lower than in group I (*p* < 0.05). IL-1 level in groups IV and V decreased by 30.47 and 24.74% (*p* < 0.05), respectively, compared with group I, with no significant difference (p > 0.05) between groups IV and II. Similarly, group II showed the lowest IL-6 level (2.02 mg/g), which was significantly lower than in group I (*p* < 0.05). IL-6 level in groups IV and V decreased by 28.17 and 23.53% (*p* < 0.05), respectively, compared with group I, with no significant difference (p > 0.05) between groups IV and II. Notably, group IV showed the highest IL-10 level (3.84 mg/g), which was significantly higher than in groups I, II, and III (*p* < 0.05). In addition, IL-10 level in groups IV and V increased by 45.45 and 36.36% (*p* < 0.05), respectively, compared with group I. Group II showed the lowest TNF-*α* level (2.66 mg/g), which was significantly lower than in other groups. Furthermore, TNF-α level in group IV decreased by 25.49% (*p* < 0.05) compared with group I. These results indicate that though gentamicin effectively suppresses inflammatory factors, it also reduces immune activity. In contrast, SBWP supplementation enhanced immune function while simultaneously reducing inflammation.

**Table 6:** Effect of SBWP on serum immune indexes of newborn calves.

Group	Group I	Group II	Group III	Group IV	Group V
IgG (μg/mL)	11.91 ± 4.65a	10.76 ± 1.69a	14.18 ± 4.13a	18.10 ± 2.32b	17.79 ± 3.32b
IL-1 (pg/mL)	3.84 ± 0.12d	2.45 ± 0.14a	3.34 ± 0.02c	2.67 ± 0.53ab	2.89 ± 0.32b
IL-6 (pg/mL)	3.23 ± 0.10c	2.02 ± 0.35a	3.17 ± 0.02c	2.32 ± 0.53ab	2.47 ± 0.32b
IL-10 (pg/mL)	2.64 ± 0.14b	2.24 ± 0.32a	3.23 ± 0.17c	3.84 ± 0.34d	3.60 ± 0.26d
TNF-α (pg/mL)	4.59 ± 0.27d	2.66 ± 0.49a	4.19 ± 0.26c	3.42 ± 0.16b	3.67 ± 0.63b
IFN-γ (pg/mL)	3.82 ± 0.02	3.41 ± 0.08	3.71 ± 0.20	3.32 ± 0.34	3.43 ± 0.16

### 3.4 Effect of SWBP on four species of fecal bacteria of newborn calves

**Figure 1:**
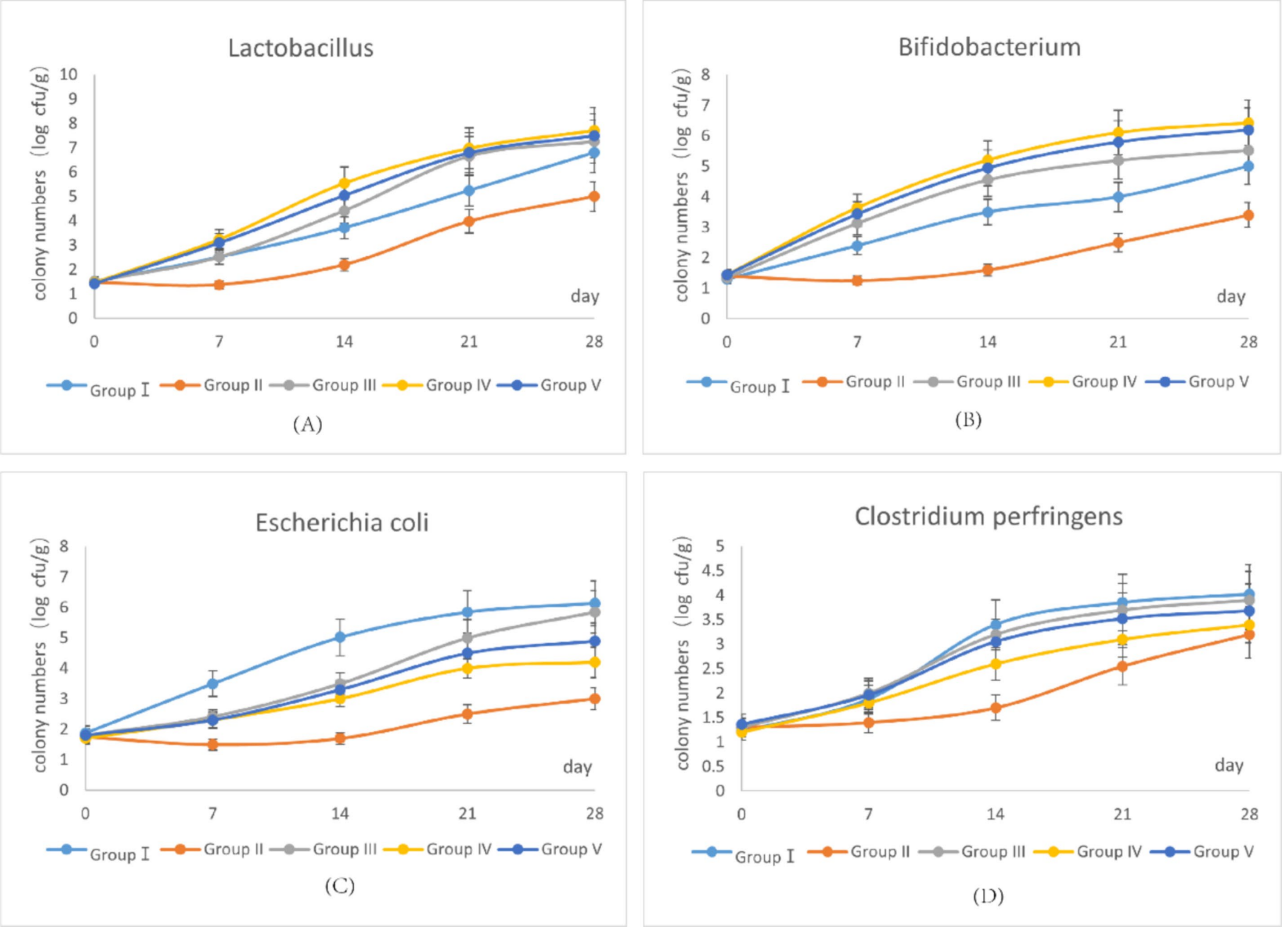
Effects of SBWP on fecal microorganisms in newborn calves. **(A)** Variation curve of *Lactobacillus* in fecal samples with the age of calves. **(B)** Variation curve of *Bifidobacterium* in fecal samples with the age of calves. **(C)** Variation curve of *Escherichia coli* in fecal samples with the age of calves. **(D)** Variation curve of *Clostridium perfringens* in fecal samples with the age of calves.

The dynamics of four predominant fecal bacterial populations over the trial period are represented in Figure 1. As shown in Figures 1A, B, the growth trends of *Lactobacillus* and *Bifidobacterium* in group IV were higher than in group I. By the end of the trial, group IV showed the highest abundance of *Lactobacillus* and *Bifidobacterium*, whereas group II showed the lowest. In contrast, as shown in Figures 1C, D, the growth trends of *E. coli* and *C. perfringens* were highest in group I. By the end of the trial, group I showed the highest abundance of these pathogens, whereas group II showed the lowest. Notably, the growth of *E. coli* and *C. perfringens* in group IV was suppressed during the 0–14 trial day period compared with group I. These findings suggest that supplementation with 500 mg/d SBWP promotes the growth of beneficial bacteria (*Lactobacillus* and *Bifidobacterium*) while suppressing the growth of pathogenic bacteria (*E. coli* and *C. perfringens*). However, gentamicin supplementation suppressed the growth of all four bacterial populations.

SBWP supplementation significantly modulated fecal microbiota composition in newborn calves at the 28th trial day (Table 7). The number of *Lactobacillus* and *Bifidobacterium* species increased dose-dependently, with groups IV (7.71 ± 0.93) and V (7.50 ± 0.90) showing the highest *Lactobacillus* levels (*p* < 0.05, vs. I:6.81 ± 0.82) and groups IV (6.43 ± 0.77) and V (6.20 ± 0.74) showing the highest *Bifidobacterium* levels (*p* < 0.05, vs. I:5.00 ± 0.60). *E. coli* was suppressed in group II (3.00 ± 0.36a), but it was significantly increased in groups IV (4.20 ± 0.50b) and V (4.90 ± 0.59b) compared with group I (*p* < 0.05). Minimal fluctuations were observed in *C. perfringens* (3.20 ± 0.48a–4.02 ± 0.60b), with group IV showing a significantly decreased level compared with group I (*p* < 0.05). These results highlight the prebiotic potential of SBWP in enhancing the growth of beneficial bacteria while transiently regulating pathogens.

**Table 7:** Effects of SBWP on fecal microorganisms in newborn calves at the 28th trial day (log CFU/g).

Microbial species	Group I	Group II	Group III	Group IV	Group V
*Lactobacillus*	6.81 ± 0.82ab	5.00 ± 0.60a	7.25 ± 0.87b	7.71 ± 0.93c	7.50 ± 0.90c
*Bifidobacterium*	5.00 ± 0.60b	3.40 ± 0.41a	5.53 ± 0.66bc	6.43 ± 0.77c	6.20 ± 0.74c
*Escherichia coli*	6.14 ± 0.74c	3.00 ± 0.36a	5.85 ± 0.70c	4.20 ± 0.50b	4.90 ± 0.59b
*Clostridium perfringens*	4.02 ± 0.60b	3.20 ± 0.48a	3.90 ± 0.59b	3.40 ± 0.51a	3.68 ± 0.55ab

### 3.5 Effect of SWBP on fecal microbiota of newborn calves

In this study, the V3–V4 hypervariable region of the 16S ribosomal DNA (rDNA) extracted from fecal samples was amplified and subjected to high-throughput sequencing analysis using the Ion S5™ XL sequencing platform (Shanghai Baiqu Biomedical Technology Co., LTD.). Metagenomic sequencing of 18 jejunal specimens resulted in a comprehensive dataset comprising 1,416,548 high-quality sequence reads. Stringent quality control measures and bioinformatic processing were followed, and each biological replicate yielded an average of >76,000 high-fidelity bacterial 16S rDNA sequences. The asymptotic trajectory of the rarefaction curve showed that the achieved sequencing depth provided sufficient coverage of jejunal microbiota, with the curve reaching a plateau, which suggests the near-complete sampling of resident microbiota. This quantitative assessment of sequencing saturation confirms the robustness and representativeness of the acquired metagenomic data for subsequent taxonomic classification and ecological analyses of the jejunal microbiome.

The effects of SBWP on *α*-diversity indices of fecal microbial communities are presented in Table 8. Statistical analysis showed a significant decrease in Observed_species, Shannon, Simpson, Chao1, and ACE diversity indices in group II compared with group I (*p* < 0.05). Notably, 500 mg/day/calf SBWP showed a tendency to increase microbial diversity parameters, including Observed_species, Chao1, and ACE indices. These findings indicate that 0.005% gentamicin decreases microbial richness and community evenness in the fecal microbiota, while 500 mg/day/calf SBWP tends to increase the richness of the microbial structure.

**Table 8:** Effects of fecal microflora on α-diversity index.

Sample name	Group I	Group II	Group IV
Observed_species	336.66 ± 81.59b	251 ± 24.52a	379.33 ± 37.82b
Shannon index	3.91 ± 0.43b	2.68 ± 0.34a	3.78 ± 0.44b
Simpson index	0.79 ± 0.11b	0.96 ± 0.37c	0.56 ± 0.88a
Chao1 index	350.30 ± 49.94b	244.06 ± 27.51a	358.13 ± 84.15b
ACE index	336.94 ± 25.07b	243.42 ± 29.94a	384.26 ± 46.98b

**Figure 2:**
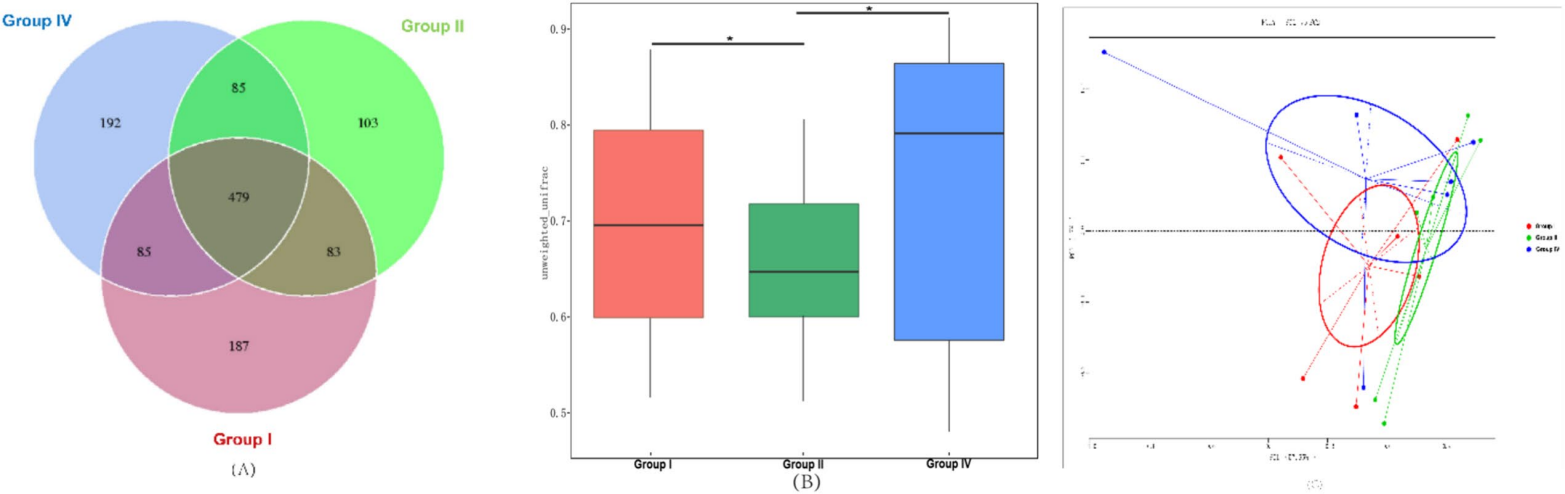
Analysis of fecal bacterial OTUs and β-diversity. **(A)** Venn diagram of OTUs of fecal bacteria with different treatments; **(B)** comparison of the β-diversity index calculated based on the unweighted UniFrac distance; **(C)** principal coordinate analysis (PCoA) of the fecal bacterial community based on the unweighted UniFrac distance at the OTU level. *, *p* < 0.05; **, *p* < 0.01, ***, *p* < 0.001.

The results of fecal bacterial OTU analyses are shown in Figure 2A. A Venn diagram constructed based on OTU clustering data revealed a total of 479 core OTUs shared between the three experimental groups, with 187, 103, and 192 unique OTUs being identified in groups I, II, and IV, respectively. These findings suggest that the supplementation of 500 mg/day/calf SBWP and 0.005% gentamicin modulates the microbiota structure in the fecal matter of newborn calves.

Analysis of the *β*-diversity index using unweighted UniFrac distances showed an extremely significant reduction in the β-diversity index in group II and a significant increase in the β-diversity index in group IV compared with group I (*p* < 0.05), as shown in Figure 2B. PCoA of fecal microbiota structure at the OTU level (Figure 1C) further elucidated these differences, with principal component 1 (PC1) accounting for 57.65% and principal component 2 (PC2) explaining 17.00% of the bacterial variance, as shown in Figure 2C.

**Figure 3:**
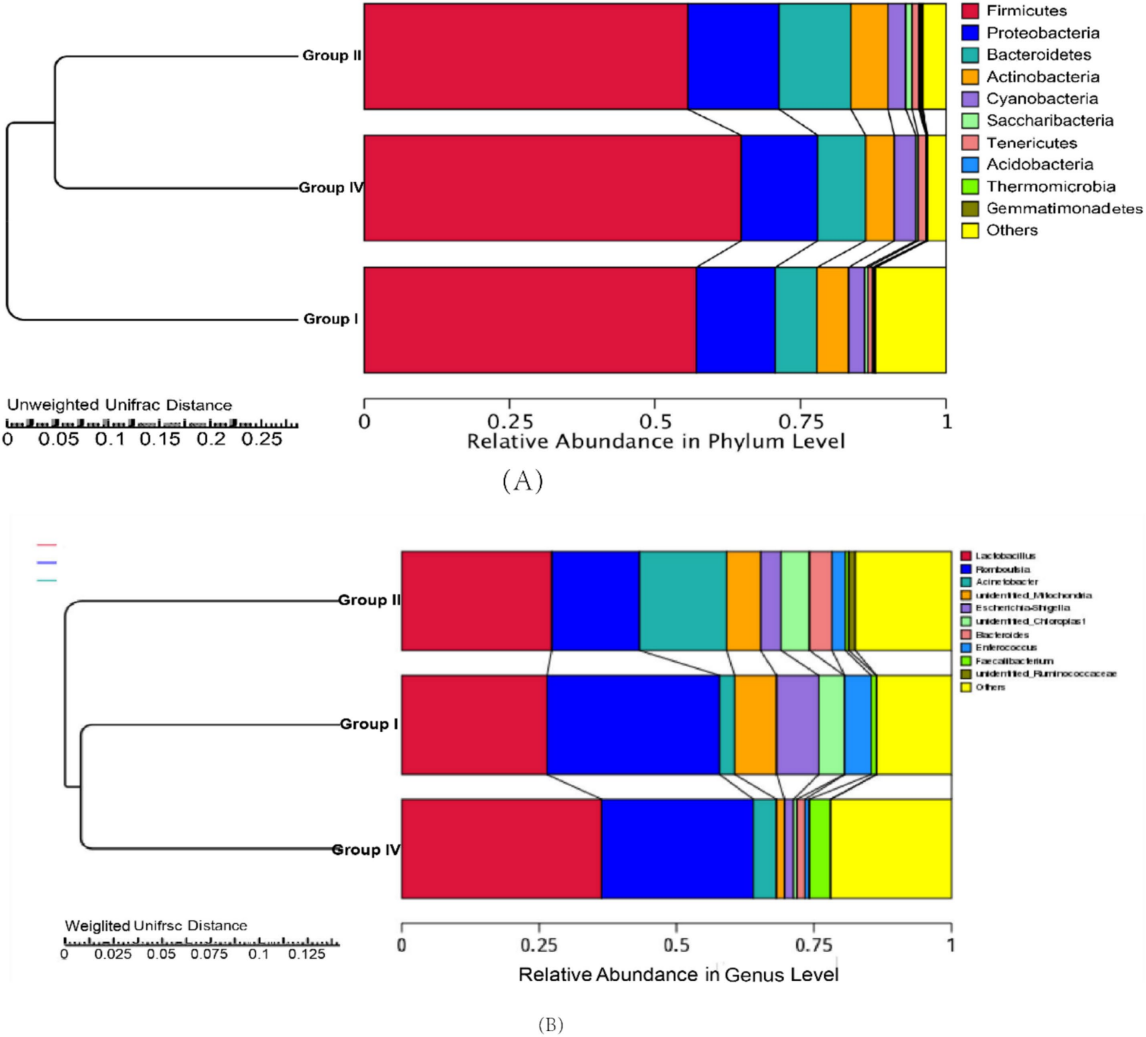
Relative abundance of fecal bacteria and MetaStats analysis of the differences at the phylum **(A)** and genus levels **(B)**.

Taxonomic composition at the phylum and genus levels is presented in Figures 3A,B, respectively. At the phylum level, the core microbiota (defined as relative abundance ≥5.0%) across groups I, II, and IV consisted of *Firmicutes*, *Proteobacteria*, *Bacteroidetes*, *Actinobacteria*, *Cyanobacteria*, and *Saccharibacteria* (Figure 3A). At the genus level, *Lactobacillus*, *Romboutsia*, *Acinetobacter*, *unidentified_Mitochondria*, *Escherichia-Shigella*, and *unidentified_Chloroplast* were identified as the predominant genera (Figure 3B). Notably, 500 mg/day/calf SBWP showed a distinct microbial composition, characterized by an increased relative abundance of *Firmicutes* and *Lactobacillus* concomitant with the decreased abundances of *Escherichia-Shigella*, *unidentified_Chloroplast*, and *Enterococcus*. In contrast, 0.005% gentamicin decreased the relative abundance of *Romboutsia* and *Escherichia-Shigella* but increased the relative abundance of *Acinetobacter* and *Bacteroides*.

The impact of SBWP on fecal microbiota was analyzed at the phylum and genus taxonomic levels, as shown in Tables 9, 10. At the phylum level, the predominant microbial communities, ranked by relative abundance, comprised *Firmicutes, Proteobacteria, Cyanobacteria, Bacteroidetes, Actinobacteria, Verrucomicrobia, Tenericutes, Fusobacteria, Acidobacteria,* and *Saccharibacteria*. In addition, comparative analysis revealed that group II showed a statistically significant decrease (*p* < 0.05) and increase (*p* < 0.05) in the relative abundance of *Firmicutes* and *Bacteroidetes*, respectively, compared with group I. Furthermore, group IV showed a statistically significant increase (*p* < 0.05) in the relative abundance of *Bacteroidetes*, compared with group I. Notably, 500 mg/day/calf SBWP showed a tendency to increase the relative abundance of *Firmicutes* while decreasing *Proteobacteria*. In contrast, 0.005% gentamicin increased the relative abundance of *Proteobacteria.*

**Table 9:** Effects of fecal microflora at the phylum level (%).

Item	Group I	Group II	Group IV
*Firmicutes*	80.69 ± 15.31b	63.87 ± 8.27a	86.71 ± 6.06b
*Proteobacteria*	13.31 ± 13.05ab	23.42 ± 10.46b	7.97 ± 4.09a
*Cyanobacteria*	4.66 ± 10.14	5.20 ± 11.27	0.94 ± 8.89
*Bacteroidetes*	0.32 ± 0.04a	4.95 ± 0.12b	2.11 ± 0.30b
*Actinobacteria*	0.56 ± 0.01	2.01 ± 0.03	0.12 ± 0.01
*Verrucomicrobia*	0.20 ± 0.4	0	0
*Tenericutes*	0.03 ± 0.02	0.31 ± 0.04	0.13 ± 0.03
*Fusobacteria*	0	0.01 ± 0.01	0.03 ± 0.04
*Acidobacteria*	0	0	0.04 ± 0.02
*Saccharibacteria*	0.03 ± 0.01	0	0.04 ± 0.01
*Others*	0.20 ± 0.18	0.16 ± 0.29	1.90 ± 0.45

**Table 10:** Effects of fecal microflora at the genus level (%).

Item	Group I	Group II	Group IV
*Lactobacillus*	27.33 ± 4.29a	28.78 ± 12.62a	66.36 ± 9.61b
*Romboutsia*	38.89 ± 24.87c	17.01 ± 1.76a	31.86 ± 10.90b
*Acinetobacter*	2.76 ± 3.40a	15.83 ± 14.68b	4.23 ± 3.03a
*Unidentified_Mitochondria*	7.64 ± 15.13	6.19 ± 10.90	1.46 ± 1.37
*Escherichia-Shigella*	0.56 ± 0.14b	0.25 ± 0.11a	0.35 ± 0.06a
*unidentified_Chloroplast*	4.62 ± 10.13	5.16 ± 11.27	0.62 ± 0.85
*Bacteroides*	0.08 ± 0.08	4.08 ± 9.38	1.45 ± 2.25
*Enterococcus*	4.83 ± 7.71	2.42 ± 1.35	0.8 ± 0.89
*Faecalibacterium*	0.96 ± 2.23	0.65 ± 0.84	3.87 ± 6.57
*Unidentified_Ruminococcaceae*	0.03 ± 0.07	1.18 ± 2.86	0.03 ± 0.04
*Others*	13.62 ± 19.04	17.46 ± 29.21	21.95 ± 10.80

At the genus level, the microbiota composition was dominated by *Lactobacillus, Romboutsia, Acinetobacter, unidentified_Mitochondria, Escherichia-Shigella, unidentified_Chloroplast, Bacteroides, Enterococcus,* and *unidentified_Ruminococcaceae*. Notably, group IV showed a significantly increased relative abundance of *Lactobacillus* (*p* < 0.05) compared with group III, while showing a marked decrease in the relative abundance of *Romboutsia* and *Escherichia-Shigella* (*p* < 0.05). In contrast, group II showed a significantly decreased relative abundance of *Romboutsia* and *Escherichia-Shigella*, but the relative abundance of *Lactobacillus* was not significantly affected. Furthermore, no significant difference was observed in the relative abundance of *Escherichia-Shigella* between the 0.005% gentamicin and 500 mg/day/calf SBWP groups. These findings suggest that SBWP supplementation induces significant alterations in the fecal microbiota structure of newborn calves.

## 4 Discussion

### 4.1 Effect of SWBP on growth performance and diarrhea incidence in newborn calves

The adverse effects of environmental stressors, including high wind velocity, low ambient temperature, significant diurnal temperature fluctuations, and suboptimal management practices, on neonatal calf health are major concerns for the local livestock industry. In newborn calves, a well-established correlation exists between rearing conditions, weaning-associated stress, gastrointestinal dysfunction, immunosuppression, diarrhea incidence, and growth performance, particularly in regions such as Xinjiang, China, which experiences pronounced seasonal temperature variations. In newborn calves, diarrhea is associated with high mortality rates, reduced ADG, and increased FCR due to intestinal mucosal damage, delayed intestinal development, compromised immune function, and increased bacterial translocation. Furthermore, these effects can have long-term implications on the growth performance and productivity of adult cattle.

According to data from the local Animal Husbandry Bureau, 63.50% of newborn calves experience diarrhea, with a mortality rate higher than 32.40% observed between January and April. Therefore, mitigating diarrhea incidence is crucial for reducing neonatal calf mortality. Q. Lu et al. showed that supplementation with *S. boulardii* significantly reduced fecal scores and diarrhea incidence in newborn calves during the first 21 days postpartum (*p* < 0.05) (12). Similarly, Yi Zhou et al. reported that dietary supplementation with 75 mg/kg/d of a probiotic significantly enhanced ADG while reducing fecal scores and FCR in weaned calves (13). In addition, Dong et al. (14) found that supplementation with 1 g/(head·d) of yeast cell wall polysaccharides significantly improved apparent digestibility and ADG without increasing ADFI in newborn calves. Our results are consistent with these findings, showing that supplementation with 500 mg/d/calf SBWP significantly enhanced ADG and reduced FCR without altering feed intake. Moreover, it significantly reduced fecal scores and diarrhea incidence, with effects comparable to those of 0.005% gentamicin supplementation. These results suggest that SBWP supplementation can effectively reduce diarrhea incidence and mortality in newborn calves and serve as a viable alternative to antibiotic use.

### 4.2 Effect of SWBP on immune function in newborn calves

The intestinal mucosal immune system acts as the primary defense mechanism against exogenous antigens and is crucial for maintaining animal health and performance. SBWP modulates intestinal mucosal immunity by regulating the transcription and expression of cytokine and immunoglobulin genes in intestinal epithelial and immune cells via pattern recognition receptors and immune signaling pathways. F.B.D. Laguna et al. reported that supplementation with *S. boulardii* CNCM I-1079 reduces inflammatory responses and enhances intestinal mucosal barrier integrity following vaccination (15). Heavy et al. (16) showed that *S. boulardii* supplementation improves pharmacodynamic parameters, including serum cytokine profiles and histological inflammation scores, in a murine colitis model. Borders et al. found that dietary supplementation with yeast-derived *β*-glucan significantly increases IgM and alkaline phosphatase levels in the intestinal mucosa of calves (17). Consistent with these studies, our results indicate that SBWP supplementation significantly increases serum IgG levels, which suggests enhanced innate immunity. Moreover, SBWP supplementation significantly reduced the serum levels of pro-inflammatory cytokines (IL-1, IL-6, IFN-*γ*, and TNF-*α*), which indicates its anti-inflammatory and immunomodulatory effects. Among the tested SBWP concentrations, 500 mg/d/calf was the most effective in enhancing immune function and reducing systemic inflammation.

SBWP, enriched with mannose and β-glucan, modulates intestinal health through PAMP-mediated interactions with host immune receptors. The mannose component binds to CLRs, such as the MR (CD206) on intestinal epithelial cells, which enhances mucosal barrier integrity by upregulating tight junction proteins (e.g., occludin, ZO-1) and stimulating mucin secretion (3, 10). Simultaneously, β-glucan activates dectin-1 receptors on dendritic cells, priming Th1/Th17 immune responses via the SYK/NF-κB pathway, which is crucial for pathogen clearance and IL-10-mediated anti-inflammatory signaling (4, 11). These immunomodulatory effects are in line with our observed increases in serum IgG and IL-10 levels, coupled with reductions in the levels of pro-inflammatory cytokines (IL-1β, IL-6, and TNF-α).

### 4.3 Effect of SWBP on four species of fecal bacteria on newborn calves

The establishment and maintenance of intestinal microbial homeostasis are important factors that influence animal health, particularly in newborn calves. Intestinal microbiota and their metabolites interact extensively with host physiological processes and regulate nutrient absorption, metabolism, and immune function. As a prebiotic, SBWP is metabolized by beneficial bacteria such as *Bifidobacterium* and *Lactobacillus*, which promotes their growth and proliferation. This enhances the intestinal microbial community structure and antimicrobial capacity through competitive exclusion of pathogenic bacteria. Previous studies have shown that yeast-derived mannan significantly increases *Bifidobacterium* abundance and improves microbial community structure in turkeys (18). He et al. (19) reported that supplementation with 0.3% yeast cell wall polysaccharides increases *Bifidobacterium*, *Lactobacillus*, and total bacterial counts while reducing *E. coli* and *Salmonella* populations in calf intestines. Yan et al. (20) observed similar effects, with yeast cell wall polysaccharides increasing *Bifidobacterium* population and reducing *E. coli* and *Salmonella* populations in chicken colons. In line with these findings, our study showed that 500 mg/d/calf SBWP supplementation increased *Bifidobacterium* and *Lactobacillus* populations while reducing *E. coli* and *C. perfringens* populations in neonatal calf feces. These results indicate that SBWP promotes the establishment of healthy intestinal microbiota and improves microbial community structure, with 500 mg/d/calf being identified as the optimal dosage. In contrast, 0.005% gentamicin supplementation suppressed *Lactobacillus* and *Bifidobacterium* growth, which potentially disrupted intestinal microbiota establishment and host–microbiota interactions, thereby delaying intestinal development.

SBWP serves as a fermentable substrate for commensals such as *Lactobacillus* and *Bifidobacterium* and promotes their proliferation through competitive exclusion of pathogens. Mannose residues competitively inhibit pathogenic adhesion by mimicking epithelial glycoprotein binding sites, thereby reducing *E. coli* and *C. perfringens* colonization (6, 18). Furthermore, *β*-glucan-derived short-chain fatty acids (SCFAs) reduce luminal pH, thus creating an inhospitable environment for acid-sensitive pathogens while enhancing nutrient absorption—a plausible explanation for the improved F/G ratio and ADG observed in group IV.

### 4.4 Effect of SWBP on fecal microbiota in newborn calves

Insights into the effects of SBWP on microbiota structure and diversity in the fecal samples of newborn calves were obtained from high-throughput sequencing of the 16S rRNA gene. *α*-Diversity, which was assessed using indices such as Chao1, ACE, Shannon, and Simpson, reflects microbial richness and diversity. Shannon and Simpson indices indicate community diversity and evenness, with a higher Shannon value denoting greater diversity and evenness and a higher Simpson value indicating dominant colonies and lower evenness. β-Diversity highlights the differences in microbial diversity between groups, which is analyzed using PCoA. In this study, 0.005% gentamicin supplementation significantly reduced α-diversity indices of the fecal microbiota, whereas 500 mg/day/calf SBWP supplementation showed a tendency to enhance α-diversity, albeit without reaching statistical significance. Consequently, it can be deduced that 500 mg/day/calf SBWP is markedly more effective than 0.005% gentamicin in modulating the richness and evenness of the fecal microbiota in neonatal calves. Furthermore, the unique OTUs and β-diversity indices were increased and decreased in the 500 mg/day/calf SBWP and 0.005% gentamicin groups, respectively. This observation suggests that SBWP facilitates the structural integrity and diversity of the fecal microbiota in neonatal calves, whereas gentamicin induces adverse effects. Moreover, 500 mg/day/calf SBWP supplementation led to a significant augmentation in the relative abundance of the probiotic genus *Lactobacillus* and a concomitant reduction in the relative abundance of the pathogenic taxa *Escherichia-Shigella* in the fecal microbiota. In contrast, though the addition of 0.005% gentamicin significantly decreased the relative abundance of *Escherichia-Shigella,* it did not confer a statistically significant beneficial impact on the relative abundance of *Lactobacillus*. These results are consistent with prior findings in this study. Notably, no significant disparity was observed in the relative abundance of *Escherichia-Shigella* between the 500 mg/day/calf SBWP and 0.005% gentamicin groups. This underscores the superior efficacy of SBWP over gentamicin in regulating the gut microbiota architecture of neonatal calves. Specifically, SBWP not only significantly reduces the relative abundance of the pathogenic *Escherichia-Shigella* but also markedly enhances the relative abundance of the beneficial *Lactobacillus,* thereby fostering a more diverse and balanced gut microbiota ecosystem. These findings indicate that SBWP increases probiotic populations while reducing pathogenic bacteria, thereby improving the intestinal microbiota structure.

Although both SBWP (500 mg/day/calf) and gentamicin (0.005%) reduced diarrhea incidence comparably (*p* > 0.05), the differences in their mechanisms underscore distinct advantages and trade-offs. Gentamicin, a broad-spectrum antibiotic, indiscriminately suppressed pathogenic and commensal bacteria, as evidenced by low *α*-diversity indices (Shannon and Chao1) and reduced *Lactobacillus* abundance. This non-selective suppression may delay gut microbiota maturation, impair mucosal immunity, and predispose calves to secondary infections, a well-documented drawback of prophylactic antibiotic use (5, 21) In contrast, SBWP showed targeted modulation: it enhanced *Lactobacillus* and *Bifidobacterium* populations while selectively inhibiting *Escherichia-Shigella*, effects that are attributable to its prebiotic specificity and lack of bactericidal activity. Notably, SBWP preserved *β*-diversity and increased unique OTUs, reflecting a resilient microbial ecosystem. Most importantly, SBWP achieved pathogen suppression comparable to that of gentamicin (*Escherichia-Shigella*: *p* > 0.05) without compromising microbial richness, which highlights its potential as a sustainable alternative.

## 5 Limitations and future directions

This study has several limitations. First, the 28-day trial period may be insufficient to capture the long-term effects of SBWP on postweaning performance or microbial stability. Second, the use of single geographical origin (Xinjiang) and breed (Simmental×Yili Brown) limits generalizability to other climates or genotypes.

Future research should prioritize longitudinal studies to assess the effects of SBWP on carcass traits and immune memory in adult cattle. Mechanistic investigations, such as transcriptomic profiling of intestinal epithelia and metatranscriptomic analysis of microbial function, could elucidate the role of SBWP in SCFA production and barrier reinforcement. Furthermore, combining SBWP with probiotics or phytogenics may result in synergistic benefits, which warrants further exploration.

## 6 Conclusion

The findings of this study showed that supplementation with 500 mg/d/calf SBWP reduces diarrhea incidence and enhances growth performance in newborn calves by improving immune function, reducing systemic inflammation, and optimizing fecal microbiota composition. It significantly improved the β-diversity indices and showed a tendency to increase *α*-diversity in the fecal microbiota of newborn calves, in contrast to the adverse effects induced by 0.005% gentamicin. Specifically, 500 mg/day/calf SBWP significantly increased the relative abundance of *Lactobacillus* and decreased the relative abundance of *Escherichia-Shigella*. Furthermore, the difference in the relative abundance of *Escherichia-Shigella* between the 500 mg/day/calf SBWP and 0.005% gentamicin groups was not statistically significant. In the Xinjiang region, 500 mg/d/calf SBWP showed an efficacy comparable to that of 0.005% gentamicin in enhancing serum immune factors and modulating fecal microbiota. The ability of 500 mg/d/calf SBWP to promote beneficial microbial populations, reduce pathogenic bacteria, and support immune and growth performance underscores its potential as a sustainable alternative to traditional antimicrobial agents in livestock management. Given the increasing emphasis on environmentally sustainable feed practices, green cultivation, and healthier food production, SBWP holds significant potential for application in ruminant production systems.

## Data Availability

The raw data supporting the conclusions of this article will be made available by the authors, without undue reservation.
